# Selectivity of Face Perception to Horizontal Information over Lifespan (from 6 to 74 Year Old)

**DOI:** 10.1371/journal.pone.0138812

**Published:** 2015-09-23

**Authors:** Valérie Goffaux, Aude Poncin, Christine Schiltz

**Affiliations:** 1 Institut de recherche en sciences psychologiques (IPSY), Université Catholique de Louvain (UCL), Louvain-la-Neuve, Belgique; 2 Institute of Neuroscience (IONS), Université Catholique de Louvain (UCL), Louvain-la-Neuve, Belgique; 3 Cognitive Neuroscience Department, Maastricht University, Maastricht, The Netherlands; 4 Institute of Cognitive Science and Assessment (COSA); Education, Culture, Cognition and Society (ECCS) unit, University of Luxembourg, Walferdange, Luxembourg; University of British Columbia, CANADA

## Abstract

Face recognition in young human adults preferentially relies on the processing of horizontally-oriented visual information. We addressed whether the horizontal tuning of face perception is modulated by the extensive experience humans acquire with faces over the lifespan, or whether it reflects an invariable processing bias for this visual category. We tested 296 subjects aged from 6 to 74 years in a face matching task. Stimuli were upright and inverted faces filtered to preserve information in the horizontal or vertical orientation, or both (HV) ranges. The reliance on face-specific processing was inferred based on the face inversion effect (FIE). FIE size increased linearly until young adulthood in the horizontal but not the vertical orientation range of face information. These findings indicate that the protracted specialization of the face processing system relies on the extensive experience humans acquire at encoding the horizontal information conveyed by upright faces.

## Introduction

The visual mechanisms disrupted by face inversion have been extensively investigated, as they presumably account for what makes (upright) face perception special. Evidence indicates that human adults encode upright faces more globally than inverted faces (e.g., [1]). It is indeed far more difficult to judge one facial feature (e.g., eyes) without being influenced by the other features (e.g., nose, mouth, chin, etc.) in upright than inverted faces [2–5]. For that reason upright faces are said to be processed ‘holistically’, or ‘interactively’ in contrast to inverted faces, which are processed more locally [1]. The so-called whole-part, composite and congruency tasks systematically measure face processing interactivity. The face inversion effect (FIE) is also taken to reflect the interactivity of upright face processing and the engagement of face-specialized processes in general [1].

**Fig 1:**
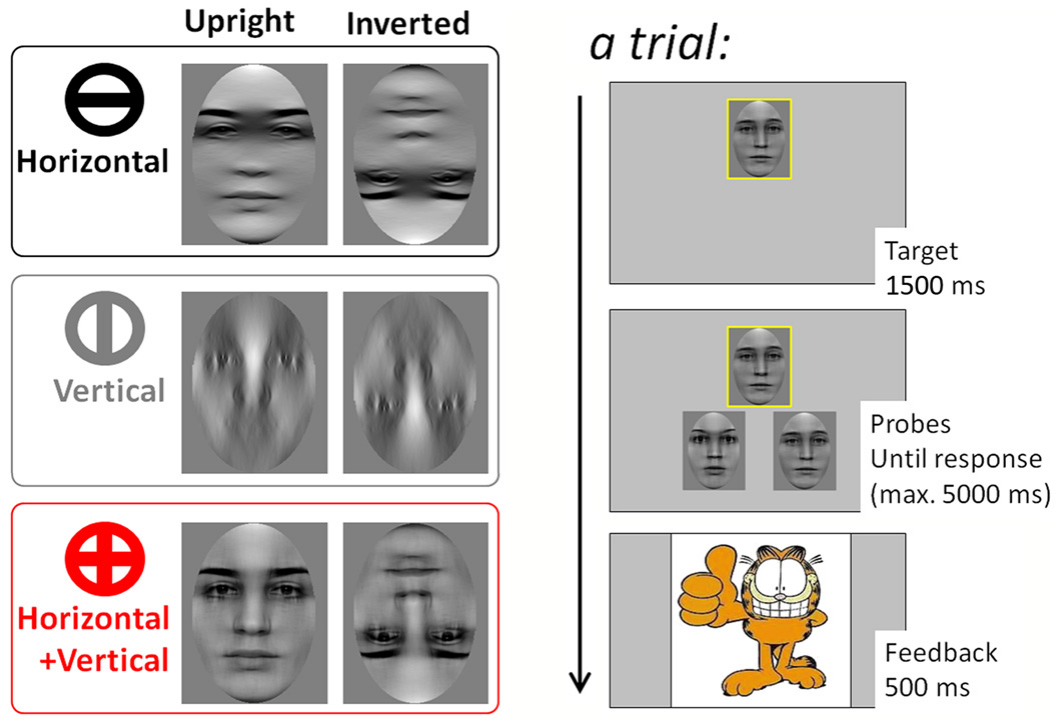
Task and stimuli. Left. Example of a face stimulus filtered to preserve either horizontal (H), vertical (V), or both information (HV). Right. Trial temporal structure.

Recent work revealed that the mechanisms specifically engaged for the processing of upright faces are driven by horizontal information (see Fig 1). Dakin and Watt [6] filtered naturalistic pictures of celebrities to preserve information in nine narrow orientation ranges centered on vertical to horizontal orientation in steps of 22.5 degrees. Recognition performance of adult observers peaked for horizontally-filtered images; it declined for oblique and reached its minimum in the vertical range. The fact that face images convey more energy in the horizontal compared to other orientation ranges [7] does not fully account for the horizontal advantage of facial identity processing. Goffaux and Dakin (2010) indeed showed that the horizontal advantage for facial identity processing applies only when viewing upright faces [8]. For inverted faces identity processing accuracy is comparable in the horizontal and vertical ranges of face information. By means of an ideal observer approach, Pachai and colleagues [9] further confirmed that the FIE relates to the inability to encode horizontal information in inverted faces. Horizontal advantage is therefore viewed as reflecting the biases of the mechanisms that are recruited for the specialized processing of upright faces. In line with this notion, Goffaux and Dakin (2010) reported that interactive congruency effects, taken to mark the engagement of face-specialized mechanisms, are robust based on horizontal but not vertical information.

Altogether these findings suggest that the visual information most crucial for the specialized processing of faces in the adult human brain resides in the horizontal range of orientation. This evidence offers perspectives for a parsimonious description of the information driving face-specific processing that is directly grounded in the primary decomposition of the visual information in V1 [10]. The present research addressed when the face processing system preferentially tunes to horizontal information over the human development. Is horizontal tuning an invariable processing bias? Or is modulated by the extensive experience that humans acquire with faces over the lifespan? To answer this question, we explored the developmental trajectory of the horizontal tuning of face-specific processing from childhood to elderly adulthood.

Early in life, newborns already show rudimentary perceptual capabilities for faces: they orient preferentially towards patterns, which, like faces, contain more elements in the upper than the lower part [11–14] and they prefer to look at upright compared to inverted faces (e.g., [15]). The determinants of the newborn preference for faces quickly change to become more specific to the faces encountered in the natural visual environment. Chien (2011) indeed reported that 2 month-old infants spend more time looking at naturalistic face photographs than at top-heavy non-face stimuli [16]. Infants also start to prefer looking at “own-race” and own-species faces [17, 18].

Despite face perception abilities at birth, children still process faces less proficiently than adults (e.g, [19]). Carey and Diamond [20] showed that accuracy in a match-to-sample task improves by 20% from 6 to 10 years of age. Germine et al. (2011) recently reported that face recognition performance further improves by 18% from 10 to 30 years of age [21]. Some authors propose that the improvement of face processing over development is due to the maturation of general cognitive abilities (concentration, attention, use of strategies, visual acuity, memory, etc. [22, 23]). The face-specific visual mechanisms are, according to these authors, fully mature at 5 years of age. Conversely, other authors argue that developmental improvements in face perception are due to the maturation of specialized mechanisms [24–28], which culminates at adolescence [29] or even at adulthood [21, 30]. Some reports systematically demonstrated that face processing improvements during childhood cannot be explained by general cognitive development [28, 31] but reflects the fine-tuning of interactive mechanisms [25, 29, 32–35], particularly in the eye region [28].

After age 30, face recognition declines [36–40]. The reason of this deterioration is still poorly understood. Some studies reported a lesser sensitivity to feature spatial arrangement [41, 42]. In contrast, Boutet and Faubert (2006) reported comparable FIE size across young and elderly adults, suggesting intact interactive processing in the latter population [43]. However, studies directly exploring interactive processing reported very mixed results. Boutet and Faubert (2006) found no composite illusion in older adults whereas the whole-part advantage was as strong in young as in elderly adults. Konar et al. (2013) also studied the composite illusion in elderly adults and observed that the deterioration in face recognition in this population goes along with an increased reliance on interactive processing. This agrees with the findings of Germine et al. (2011) that FIE increases until at least 65 years of age [21].

Altogether these findings suggest that core aspects of face-specific processing (i.e., interactive processing, FIE) continue to develop until at least adolescence. However the exact nature of the face information that humans learn to better extract over the lifespan is not clear.

The present study addressed this question by investigating the orientation range underlying the development of face perception from childhood to elderly adulthood. Participants aged between 6 and 74 years were tested for their ability to match unfamiliar faces that were filtered to selectively retain information in narrow ranges centered on horizontal (H), vertical (V), or both orientations (HV). We used a semi-delayed matching task designed to minimize general cognitive demands and to be readily performed by subjects from 6 to 74 year-old. Filtered faces were presented at upright and inverted planar orientations in order to evaluate the developmental course of the FIE and its relationship with the participants’ sensitivity to horizontal information. H and V conditions were chosen because these orientation ranges are supposed to contain the cues that maximally and minimally activate specialized mechanisms for the processing of facial identity, respectively. We included the combined HV condition because it (1) resembles the broadband stimuli encountered in everyday-life visual environment and (2) is the sum of the H and V conditions of interest, therefore offering an ideal comparison standard.

Using FIE to evaluate face-specific processing has several advantages. First, the matching of upright and inverted faces is expected to involve comparable general cognitive mechanisms. Second, upright and inverted faces are well matched at the level of the intrinsic properties of the physical signal they convey therefore excluding accounts in terms of general visual processes (e.g. pattern encoding, visual memory, etc.). A difference in favor of upright faces is therefore taken to reflect the recruitment of observer-dependent mechanisms specifically at work while viewing upright faces.

We hypothesized that if face perception matures due to the refinement of face-specialized mechanisms that preferentially rely on horizontal information, FIE should increase with age for the processing of horizontal face information (i.e., in H condition). In contrast, FIE should be relatively small and stable over age when based on vertical face information (i.e., V condition). Alternatively if face perception development reflects the refinement of orientation integration, FIE should develop only when multiple orientation ranges are present in the stimulus (i.e., in the HV condition). Last, it could be that adult horizontal tuning is prewired and that the development of face-specific processing reflects the refinement of mechanisms that are not sensitive to image orientation content; the FIE should then develop similarly in all filter conditions.

## Methods

### Subjects

We tested 296 healthy subjects (150 females) aged from 6 to 74 years (see Table 1). All subjects were recruited and tested in Luxembourg. They had normal or corrected-to-normal visual acuity as confirmed by their results on a computerized version of the Landolt test performed before the experiment.

**Table 1:** Sample details per age group.

Age (years)	Mean	*Std*	N outliers	N males	Total n
6–7	6.4	.*50*	2	11	24
8–9	8.5	.*51*	3	15	24
10–11	10.3	.*46*	3	27	58
12–13	12.6	.*49*	1	11	31
14–15	14.4	.*50*	1	22	43
16–17	16.4	.*50*	3	15	30
18–19	18.4	.*49*	1	15	29
20–35	25.8	*5*.*04*	0	12	22
60–74	65.7	*4*.*28*	0	11	21
**Total**			**14**	**139**	**282**

The experimental protocol of our study adhered to the declaration of Helsinki, APA ethical standards for research and national regulations. Prior to participation, our participants were indeed informed about the purpose of the research and that they were free to stop their participation at any time. All participants and parents of participants aged below 12 provided their free and informed consent. It is also important to mention that our task and stimuli (i.e., matching neutral unknown faces) were specifically designed to be performed effortlessly by participants ranging from 6 to 74 years of age (the completion of the whole data collection lasted about 30 minutes). Our task and stimuli have been approved for children and adult testing by the research ethics board of Maastricht University (ECP-7-11-2004-A1 and ECP number: 7-11-2004, respectively).

Children and adolescents were recruited in primary and high schools in Luxembourg, respectively. They were tested in a quiet classroom. Due to technical issues during the encoding of subject information, we did not record the exact age (in months) of all 6-year-old and some 10-year-old children (19 out of the 41). Per default, those subjects were classified as being exactly 6 (72 months) and 10 (120 months) old. For this reason, the mean age of the 6–7 and 10–11 years age groups in Table 1 are only approximate and the respective standard deviations underestimated. This artificial reduction in age variation in the 6 and 10-year cohorts may have slightly reduced the chance for finding a significant relationship between age and performance.

Young adults were students recruited at the University of Luxembourg. Adults aged between 25 and 35 years were invited to participate via Facebook. Young adults were tested in a lab of the department.

The elderly adults were recruited and screened by their medical doctor to exclude any precursor sign of cognitive difficulties using the Montreal Cognitive Assessment test (MoCA; [44]). Participants failing to reach a score of at least 22 of 30 possible points were not included in the final sample. Consent was administered prior to their participation in the experiment. Elderly participants were tested in a room next to the MD’s office.

### Stimuli

We used forty unfamiliar greyscale pictures of faces (190 by 240 pixels; twenty-six females, fourteen males) posing in a neutral expression. Stimulus availability was the sole reason for the imbalance in stimulus gender. Models were alumni Psychology students (aged between 18 and 25 years old) of the Université Catholique de Louvain (Belgium). First we normalized face images to obtain a mean (luminance) of 0 and a root-mean square (RMS) contrast of 1. Next, images were Fast Fourier transformed and the resulting Fourier energy was multiplied with wrapped Gaussian filters (20°FWHM) centered either on H or V orientation. HV faces were constructed by summing the H and V Fourier energy of each image (as in [8]; see Fig 1). After the inverse Fourier transform, the luminance and RMS contrast of all resulting image was adjusted to match the average luminance and RMS contrast of the original image set. The output faces were further embedded in an oval-shaped aperture to eliminate external cues to facial identity (hair, neck, ears, and outer contour; Fig 1). Inverted stimuli were created by flipping each image vertically.

All testing was performed in a dimly lit room on a laptop (TFT LCD screen; screen size: 12.1”; screen resolution: 1024 by 768 pixels). All age groups were tested using the same set of four identical laptop models. Monitor parameters (luminance and resolution) were strictly matched across laptops. Moreover, the fact that our analyses are based on relative FIE measures further warrants that our results are uncontaminated by any residual screen differences. At a viewing distance of 30 cm, stimuli were presented on a light grey background (RGB values: 192, 192, 192) and subtended a visual angle of 7.2 by 8.9 degrees.

### Procedure

Participants performed an intuitive and child-friendly semi-simultaneous match-to-sample task designed to guarantee above chance-level performance in all age groups. They were individually instructed to help a cartoon character to sort (match) the pictures of cartoon friends as accurately and fast as possible. One target face picture (framed by a 5 pixel-wide yellow border) appeared at the top center of the screen for 1500 ms then two probe face pictures were shown at the left or right part of screen bottom. The screen location difference between target and probes was expected to discourage the use of pixel-level image comparisons while performing the task.

Target and probe stimuli stayed on screen until subject’s response or for a maximum duration of 5000 ms in order to minimize the time pressure for response deliverance. Participants had to match the top target face picture with one of the probe faces at the bottom of the screen. They did so by pressing on the corresponding left or right response key. The match probe appeared on the right or left side of the screen equally often. Faces were shown at upright picture-plane orientation in half of the trials and inverted in the remaining of the trials. On a given trial, all faces were of the same picture-plane orientation and filter condition.

Participants first practiced the task with cartoon images; then they were familiarized with the upright and inverted filtered face pictures, and practiced the task with 12 trials randomly selected from the list of the experimental trials. On every trial, participant’s response was immediately followed by positive or negative feedback (cartoon expressing happiness or boredom on a green or red background, respectively) for 500 ms, depending on the accuracy of their response. The next trial started immediately after the offset of the feedback screen.

The planar orientation (upright, inverted) and filter (HV, H, V) were varied randomly every 20 trials in order to minimize the cognitive load that may result from the switching between different conditions. A self-paced resting pause occurred after each block. There were 40 trials per conditions making a total number of trials of 240 experimental trials. The experiment lasted for approximately 20 minutes.

### Data analyses

Prior to statistical analysis, we screened the data to remove outliers. Standardized residuals were computed at the individual level for correct reaction times (RTs) and accuracy scores in each of the six Planar Orientation by Filter conditions (upright-HV, upright-H, upright-V, inverted-HV, inverted-H, inverted-V). Whenever the computed residuals were higher than 3 or lower than -3, for RTs and/or accuracy, the subject was excluded from the sample. Fourteen subjects in total were rejected following this procedure (see Table 1 for the numbers of outliers per age group and Table 2 for the mean and standard errors of the RT per age groups after outlier rejection).

**Table 2:** Mean and standard deviation of correct response time (in milliseconds) for each age group in each Planar Orientation by Filtering condition separately.

Age (years)	Upright-HV		Upright-H		Upright-V		Inverted-HV		Inverted-H		Inverted-V	
	Mean	*std*	Mean	*std*	Mean	*std*	Mean	*std*	Mean	*std*	Mean	*std*
6–7	1876	*308*	1902	*338*	1891	*429*	2052	*454*	1921	*428*	1894	*365*
8–9	1740	*390*	1844	*385*	1919	*367*	1835	*360*	1892	*297*	1834	*375*
10–11	1420	*293*	1518	*328*	1562	*298*	1576	*330*	1558	*324*	1559	*281*
12–13	1256	*293*	1372	*322*	1505	*308*	1445	*263*	1550	*338*	1567	*367*
14–15	1210	*376*	1260	*324*	1344	*322*	1304	*291*	1361	*331*	1453	*369*
16–17	1216	*295*	1206	*296*	1344	*324*	1339	*342*	1380	*343*	1388	*320*
18–19	1028	*232*	1149	*281*	1210	*270*	1163	*302*	1258	*334*	1240	*270*
20–35	1095	*266*	1206	*333*	1320	*298*	1389	*372*	1504	*471*	1425	*382*
59–74	1637	*399*	1751	*440*	1889	*441*	1988	*445*	1994	*462*	1928	*445*
**Total**	**1365**	***405***	**1445**	***417***	**1527**	***407***	**1531**	***437***	**1565**	***425***	**1562**	***399***

**Fig 2:**
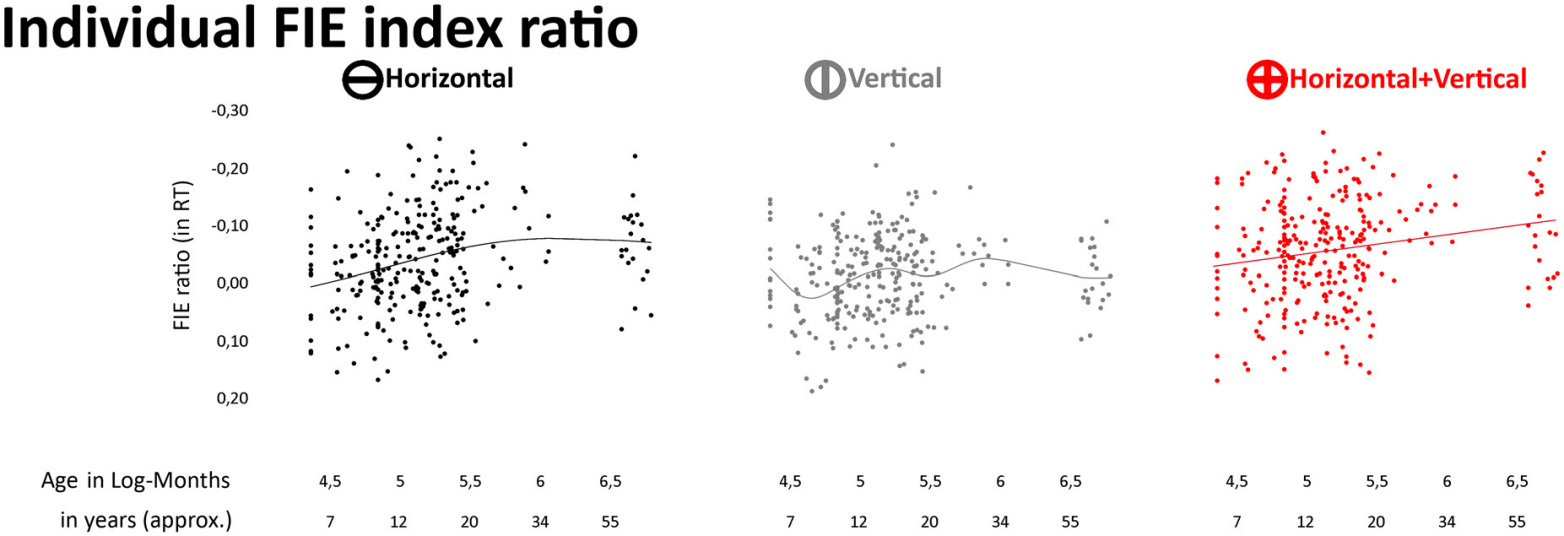
Individual FIE ratios. Individual FIE ratios (in RT) are plotted as a function of age for each Filter condition, separately. The lines depict the function best fitting FIE as a function of age. They were computed based on a Generalized Additive Model analysis [84]. In agreement with the fitting analyses, the function of FIE development was linear in HV condition and non-linear in H and V conditions.

To estimate the recruitment of face-specific processing, we computed a FIE ratio for each subject by dividing the difference between upright and inverted performance by their sum in each filtering condition (i.e., Inverted-Upright/ Inverted +Upright). FIE RT ratio indices ranged between -1 and 1, positive values indicating faster RT for upright than inverted faces (Fig 2).

Since the task was specifically designed to achieve good performance levels, accuracy was close to ceiling in several conditions and age groups (see S1 Table). The ceiling of performance shrank the range of accuracy values in some age groups, therefore leaving less room for the expression of FIE differences over development. We systematically evaluated accuracy range differences over development by means of the Levene’s test of variance homogeneity. This test confirmed heteroscedasticity (i.e., unequal variance) of the FIE accuracy ratios over age in each Filter type (Levene’s test: Fs> 3, ps< .003); in contrast, FIE RT ratios had comparable variance over development (Levene’s test: Fs< 1.7, ps> .08). For these reasons, FIE accuracy ratio were not analyzed further [22]. We only report the descriptive statistics of accuracy in a supporting table (S1 Table).

We investigated the internal (split-half) reliability of FIE RT ratios. We focused this analysis on young adult participants (aged between 20 and 35 year-old, n = 22) for sake of comparison with previous works (see below). We calculated Spearman-Brown-corrected split-half reliability of individual FIE RT ratios [45, 46] in H, HV, and V conditions separately. The p value and confidence interval were estimated via permutation tests and bootstrap, respectively (n iterations: 10,000 in each case). FIE RT ratios were reliable in each filter condition (H: r_corrected_ = .82, CI = [.78 .91], p< .0001; HV: r_corrected_ = .6, CI = [.47 .72], p< .025; V: r_corrected_ = .58, p< .03, CI = [.4 .76], p< .0001). It is interesting to note that the reliability of FIE in H condition was close to the reliability of the Cambridge Face Memory Test (CFMT), which is considered one of the most reliable measures of face recognition ability in adults [47, 48]. Unluckily, we could not find any other paper reporting FIE reliability. Few studies addressed the reliability of other behavioural markers of face interactive processing such as the composite effect. The internal reliability of the composite effect was found to range from .43 to .75 [49, 50] (see also [51]). The reliability of the present FIE RT ratios lies at the upper end of this range.

The age variable was skewed due to a denser sampling from 6 to 19 year-old than in adulthood; it also comprised a non-negligible gap between young and elderly adult groups. To minimize the distortions that these non-linearities may cause to the function relating FIE to age, we used the natural logarithm of the age expressed in month in our analyses [52].

The influence of age on the processing of H, V, and HV information on face-specific processing was explored in three different ways. First, we performed General Linear Model (GLM) analyses because they are well suited for models including quantitative (here, age) and qualitative (filter type) variables. The GLM included Filter type as a within-subject-predictor and age as a continuous between-subject predictor. We tested the hypothesis that the developmental improvements of face-specific perception relate to the enhanced encoding of horizontal (i.e., in HV and H conditions) but not vertical information

We estimated the magnitude of the reported effects and interactions using eta squared (η^2^). η^2^ quantifies the percentage of variance explained by a given factor [53].

**Fig 3:**
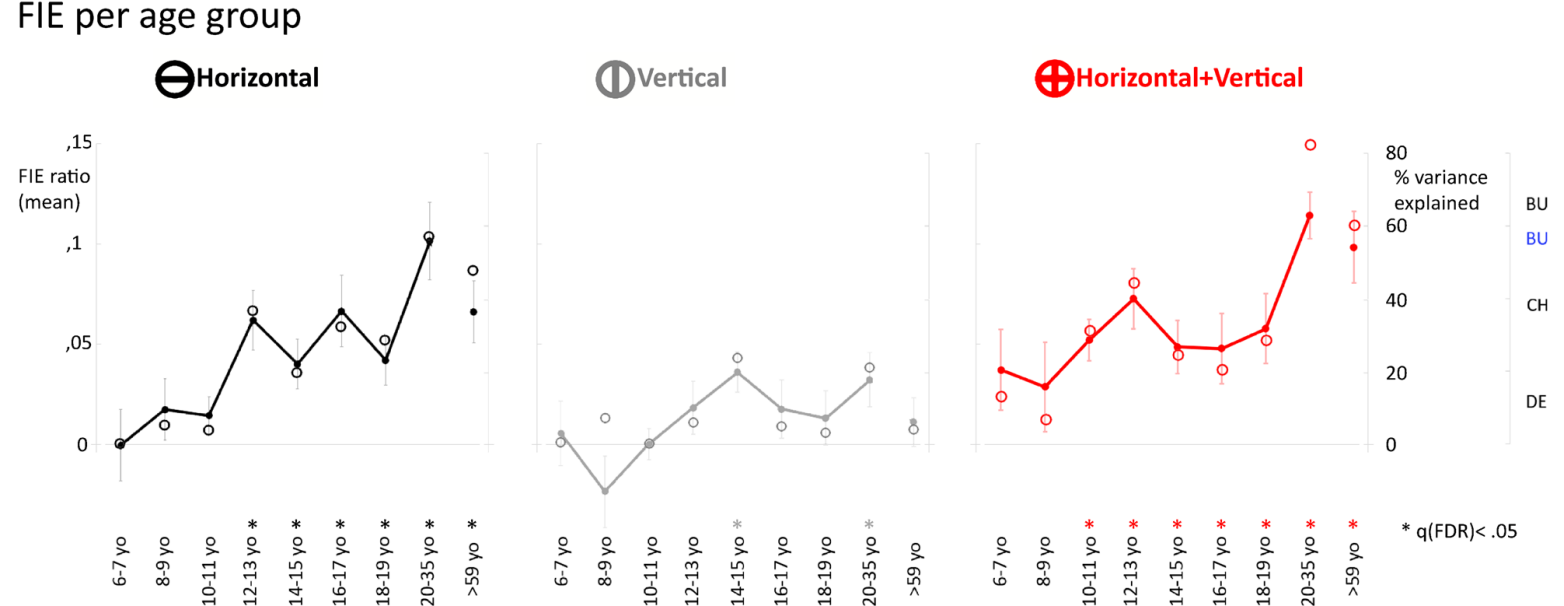
Mean and size of FIE ratio per age group. Full circles and lines illustrate the mean FIE ratio (in RT) in the different age groups separately for horizontal, vertical, and horizontal-vertical filtering conditions. Error bars represent standard errors of the means. Open circles depict FIE size (in terms of percentage of explained variance, or eta squared) for horizontal, vertical, and horizontal-vertical filtering conditions. BU, CH, and DE data points (on the right) depict the size of the FIE in RT in previous studies [42, 85, 86]. Participants were elderly adults In Busigny et al. (2011), young and elderly adults in Chaby et al. (2011), and they were children (aged between 6 and 12) and young adults in de Heering and Rossion (2012). Black BU data point illustrates FIE size in the delayed face matching task (experiment 2) of the article of Busigny and colleagues [85]; blue BU data point refers to FIE size as measured in a simultaneous face matching task (experiment 3) of the same paper. CH symbols illustrate findings of Chaby and colleagues [42] and DE symbols those of de Heering and Rossion [86]; the latter two articles used a delayed and simultaneous matching task, respectively.

Inversion was expected to slow down face matching performance, and result in positive FIE ratios. We used one-tailed t-tests in order to explore the time points where FIE significantly surpassed 0 for each filter type separately. To this aim, we split the data in nine age groups (6–7, 8–9, 10–11, 12–13, 14–15, 16–17, 18–19, 20–35, 60–74; Fig 3; see Table 1 for group composition details). We controlled for the multiplicity of performed tests using the False Discovery Rate (FDR) procedure (mafdr.m function in Matlab; [54]). Only comparisons whose q(FDR) was < .05 are reported in the results section (and on Fig 3).

Second, individual FIE ratio indices were subjected to two-tailed Pearson correlation analyses. While we hypothesized positive correlations between age and the size of FIE in H and HV conditions, we did not have any assumption about the sign of the correlation between age and FIE in the V condition. Since the FIE ratio did not only correlate with age but also across filter conditions, we computed the two-tailed Pearson partial correlation between HV FIE ratio and age when controlling for the contribution of FIE in H and/or V conditions to address whether the influence of age in the HV filter condition reflected development of either H or V processing, or their integration. Further, we computed the partial correlation between H FIE ratio and age when controlling for the contribution of FIE in the V condition to address whether the influence of age in the H condition was specific to the processing of horizontal information or whether it captured developmental influences also at stake during the processing of vertical information.

Third, we estimated whether non-linear (i.e., quadratic or cubic) polynomials fitted the development of FIE ratio better than linear function tested in the correlation analyses. The summed squares of residuals (i.e., SSE) were submitted an F test as conventionally done for model selection.

Confidence intervals of correlation coefficients and fitting parameters were computed based on bootstrap resampling procedure (n iterations = 5,000; [55]).

For sake of completeness, we present the GLM results on the RT with Planar Orientation (upright versus inverted) and Filter type (HV, H and V) as categorical within-subject predictors and age as a continuous between-subject predictor in the supporting result section (S1 Text). We also conducted partial correlation to evaluate the relationship between upright RT and age while controlling for inverted RT.

Furthermore, we report an analysis of the horizontal processing advantage in the supporting S2 Text.

## Results

Fig 2 shows the individual FIE RT ratios plotted for each Filter condition separately.

### GLM analysis of FIE

The GLM analysis with Filter type as a categorical within-subject predictor and Age as a continuous between-subject predictor revealed a main effect of Age (F(1,280) = 23.5, p< .0001, η^2^ = .03) and a marginal interaction between Age and Filter type (F(2,560) = 2.64, p = .07, η^2^ = .005). The non-significant interaction was likely due to the covariance of FIE RT ratios across HV condition on the one hand and H and V conditions on the other hand, as confirmed by sphericity test (Mauchly χ2(2) = 12.2, p < .002) and correlation analyses (see below).

Still, this marginal interaction indicated that the development of face-specific processing tends to follow different trajectories depending on the orientation content of the face image. This was confirmed by a significant interaction between Age and Filter type (F(1,280) = 3.97, p< .05, η^2^ = .0075) when we focus the GLM analysis on H and V conditions, in which facial identity processing is known to differ maximally (therefore reducing covariance between filter types).

We explored these differential developmental trends by performing separate GLM for Filter Type conditions and found a significant main effect of age only for H and HV faces (H condition: F(1,280) = 16.6, p< .0001, η^2^ = .06; HV condition: F(1,280) = 11.9, p< .001, η^2^ = .04; V condition: F(1,280) = 2.2, p = .14, η^2^ = .008).

Fig 3 shows the mean, the standard error of the means, and the size of FIE for the different age groups. We sought to determine when over the life span FIE significantly surpassed chance, in each filter condition (see Fig 3). FIE emerged first in the HV condition; it was significant from 10 years on (qs(FDR)< .01). In H condition, FIE was significant from 12 years of age on (qs(FDR)< .003). FIE in V was only sporadically significant in the 14–15 and 20–35 age groups (qs(FDR)< .025). Importantly, even when FIE was significant in V condition, its size was always substantially smaller than in HV and H conditions (Fig 3).

### Correlation and partial correlation analyses of FIE

FIE ratio indices significantly correlated with age in HV and H filtering conditions only (HV: r = .2, 95% CI = [.09 .31], p < .0007; H: r = .24, 95% CI = [.12 .34], p< .0001; V: r = .09, 95% CI = [-.03 .2], p = .14), thereby confirming the GLM finding that the encoding of H but not of V information matures over development. The correlation was positive as RT in HV and H conditions became increasingly slower for inverted compared to upright faces as a function of age (see Fig 2).

Besides a significant correlation of FIE in HV and H condition with age, we also found a significant correlation of the FIE ratio between HV and H conditions (r = .38, 95% CI = [.28 .48], p < .00001). In contrast, FIE ratio in V condition did not correlate with the other filter conditions (ps> .09).

To disentangle whether the development of FIE in HV condition reflected the development of H and/or V processing, or any extra component related to orientation integration, we ran partial correlation between HV FIE and age while controlling for H FIE, V FIE, or both. We still found a significant correlation between the FIE in HV condition and age both when controlling for FIE in H and V separately (r = .12, 95% CI = [.01 .23], p< .04 and r = .2, 95% CI = [.09 .31], p< .001, respectively) and simultaneously (r = .115, 95% CI = [.04 .22], p< .05). These results indicate that the development of the FIE in HV condition does not only reflect the separate processing of the H and V component(s) but also an extra, likely *integrative*, component.

To assess the unique contribution of horizontal processing to the development of FIE in the H condition, we further ran a partial correlation analysis between the FIE in the H condition and age while controlling for the FIE in the V condition. This correlation was significant (r = .25, CI = [.15 .34], p< .0001) therefore confirming the selectivity of the development of FIE to the processing of H information.

### Fitting FIE as a function of age

FIE development was not strictly linear: it increased in HV and H conditions from childhood to young adulthood and dropped in all filter conditions at elderly adulthood. Quadratic and cubic functions indeed accounted for the development of FIE ratio in H and V filter conditions better than linear function (linear versus quadratic: Fs(280,279)> 1.5, ps< .05; quadratic versus cubic: Fs(279,278)> 3.5, ps< .05; Fig 2). In HV condition, FIE development was best fitted by a linear function (Fs< 1.04, ps> .05). The nonlinear developmental trend of FIE ratio in H and V conditions was likely due to the decrease of FIE size from young to elderly adulthood.

## Discussion

We investigated the nature of the visual information that humans learn to encode over the life span, as the face processing system develops. Participants aged between 6 and 74 year-old matched the identity of upright and inverted face pairs. The content of face images was restricted to the horizontal and/or the vertical orientation range(s). Horizontal and vertical face information is known to respectively maximize and minimize the recruitment of face-specialized visual mechanisms, indexed here by the face inversion effect (FIE). We found that the increase in FIE size from childhood to adulthood relates to the refined extraction of horizontal information. In contrast, the processing of vertical information did not seem to develop much; FIE in this condition was indeed relatively stable over the life span. These results suggest that the specialization of the face processing system over development is tightly linked to the refined encoding of horizontal ranges of upright face information.

Face-specific processing developed both when horizontal face information was viewed in isolation or combined with vertical information. But there were striking differences between these two conditions. First, the FIE emerged earlier for faces combining horizontal and vertical information (from 10 years on) than for faces exclusively conveying horizontal information (from 12 years on). It thus seems that until 12 years, the horizontal content of face images is necessary but not sufficient to trigger specialized face processing mechanisms. It is only from 12 years on that horizontal information is enough to elicit a significant FIE. It is also at that age that the processing of upright horizontal face information is advantaged compared to the processing of upright vertical information (S2 Text). Second, the development of the FIE in the combined condition was not fully accounted for by improvements in the separate encoding of horizontal and vertical cues. The latter result suggests that, besides a progressive maturation of horizontal processing, the specialization of the face processing system depends on the improved integration of horizontal information with other orientations. The contribution of the orientation integration to face perception should be more systematically addressed in the future.

It is unlikely that the developmental effects reported here reflect the maturation of general cognitive processes or potential methodological artefacts [22, 23], for several reasons. First, we designed our matching task to minimize cognitive (mnemonic, decisional and executive) load. We also got as close as possible to daily life viewing conditions by minimizing the time pressure for response deliverance. As ceiling effects made accuracy measures unwarranted in several age groups, we analyzed RT therefore avoiding the risk of underestimating FIE size due to accuracy range compression in a subset of age groups. A second reason to exclude general cognitive or methodological accounts for our results is that developmental effects were specific to the processing of upright faces (see S1 Text) although matching upright and inverted faces recruited comparable cognitive mechanisms, with the exception of face-specific processing. Accounts in terms of general visual mechanisms are also implausible as upright and inverted face stimuli are well matched at the level of their physical properties (i.e., energy across spatial frequencies and orientation).

Studies investigating face processing from childhood to elderly adulthood using the same task and stimuli are rare [21]. Some authors proposed that the reliance on holistic mechanisms continues to increase after 60 years of age [21, 40] while others reported stable or decreased engagement of face-specific processing at older ages [41, 42]. In support of the latter view, our results indicate that irrespective of stimulus orientation content FIE size peaks between 20 and 35 years old and decreases from young to elderly adulthood. This suggests that the specialization of face perception runs until young adulthood, not later. It is however important to note that FIE still accounted for more than 62.5% of performance variance in elderly participants (72% in young adults; estimated based on the η^2^ of the main inversion effect tested using a one-way ANOVA performed per age group separately), suggesting that visual specialization for faces is still robust at older ages.

Obermeyer and colleagues (2012) recently reported that older observers process horizontal facial information less efficiently than younger adults [56]. In contrast, we found that ageing preserves the tuning of face-specific perception to horizontal orientation. This empirical discrepancy is difficult to interpret considering the radically different methodologies used in Obermeyer et al. study and ours. While the development of face-specific processing was directly targeted in our study, Obermeyer et al. (2012) instructed their participants to match facial identity across planar orientation and filtering conditions (e.g., matching an unfiltered upright face to its inverted and filtered counterpart). This non-ecological and challenging task likely confounded face-specific processing with the heavy visual extrapolation mechanisms necessarily involved [57]. It is therefore plausible that the deterioration in task performance from young to elderly adulthood reported by Obermeyer et al. (2012) reflects the deterioration of general extrapolation abilities rather than decay in face-specific processing.

It is generally assumed that inversion impairs face perception abilities [2]. However, there was a striking interindividual variability in the size and polarity of individual FIE rations plotted on Fig 3. We indeed found negative FIE RT ratios for a non-negligible proportion of our participants (24%, 32% and 44% in HV, H and V conditions respectively). This result was partly due to the immaturity of the face processing system in children. In adults (aged between 20 and 35 years), the proportion of negative FIE indeed dropped to 0% in HV condition, 18% and 27% in H and V conditions, respectively. Thorough examination of the rare face perception studies reporting individual-level FIE measures [8, 58] confirms that FIE size and polarity vary substantially across individuals. Since most studies on face perception rely on group averaging, such interindividual variability is however generally discarded [59].

Now why would face perception increasingly rely on horizontal cues over development? This is likely due to the richness of the information carried by this orientation range; it indeed conveys the features and their spatial arrangement along the axis of face elongation [7], cues which are deemed particularly important for the extraction of facial identity [8, 9, 60, 61]. Horizontal face information is also particularly tolerant against changes in viewpoint [8, 60] (see also [62]), which are amongst the main causes of face appearance alteration in natural settings. Through daily life visual experience, the face processing system would therefore naturally tune to the horizontal range of face information because it carries the most optimal and stable cues to identity.

This proposal agrees with the developmental theory that the functional specialization of visual functions is shaped by the structure of the most frequently encountered stimuli in daily life [63, 64]. Humans are most proficient at processing classes of faces they usually encounter in daily life. Besides the other -“race” and -species effects, humans also recognize faces from their own age group better than faces from other age groups. Own-age faces tend to recruit face-specific mechanisms to a larger extent than other-age faces [65–69]. Accordingly, Kuefner and colleagues [66] found a larger FIE for own- than other-age faces. In the present experiment, stimuli were pictures of young adult faces and likely restrained FIE size in children and elderly participants due to own-age bias. However, it does not account for the FIE peak in young adulthood reported here. Germine et al (2011) indeed demonstrated that FIE peaks in young adulthood irrespective of whether stimuli are children or adult faces [21]. One could still argue that the own-age bias may contribute to the developmental differences between horizontal and vertical face information processing. For example, facial age may be more accessible in horizontal than vertical band, and this would in turn produce robust own-age biases in the horizontal range and artificially inflate differences in performance across age groups. This account is however unlikely considering that the perception of facial age, unlike identity, is unaffected by inversion [70]. FIE computation is therefore expected to cancel the effect of this process. For this reason, it is unlikely that facial age processing contributed to the differential maturation of FIE in the horizontal and vertical ranges found here.

From birth to adulthood, eye region is of special interest to humans [71–76]. Recently de Heering and Schiltz (2012) reported that the development of face perception reflects the refined processing of the vertical alignment of the eye region [28]. In a related vein, the decreased sensitivity to the eye region in elderly subjects has been proposed to account for face recognition deterioration in this age range [77–79]. Since the eyes are predominantly horizontal features [7] our results may reflect the refined processing of the horizontal information selectively provided in the eye region over development.

The present study covered most of the life span, but did not explore FIE dependence on orientation before 6 years old. Studies in newborns and infants however indicate that face perception develops considerably in this age range (see reviews by [23, 80]). There are even hints in the literature that newborns may already prefer to look at horizontally- rather than vertically- filtered faces. Newborns are indeed known to be particularly sensitive to visual contrast asymmetries along the vertical axis [13]; they also prefer looking at horizontal rather than vertical gratings [81–83]. Whether these general visual biases provide the basis for the later development of the horizontal tuning of face-specific perception or whether horizontal tuning already shows some specificity to the face category around birth is currently under investigation.

In conclusion, the present findings indicate that the visual mechanisms specialized for the processing of faces are modulated over the lifespan by the extensive experience humans acquire at extracting the visual cues located in the horizontal orientation band of upright faces. The development of FIE in the combined HV condition, taken here as a proxy of broadband face stimuli encountered in everyday-life visual environment, likely reflected an extra component related to the integration of horizontal with other orientations.

## Supporting Information

S1 Table Accuracy. Accuracy means and standard deviations (in percent correct) for each age group in each Planar Orientation by Filtering condition separately.
(DOCX)

S1 Text Age influence in filter by planar orientation (upright versus inverted) conditions. (DOCX)

S2 Text Analyses of the horizontal advantage as a function of age. (DOCX)
