# Supplementary material for: Selectivity of Face Perception to Horizontal Information over Lifespan (from 6 to 74 Year Old)
Source: PLoS One. 2015 Sep 23;10(9):e0138812. doi: 10.1371/journal.pone.0138812 (PMC4580649; doi:10.1371/journal.pone.0138812)
Supplement: S1 Table — Accuracy means and standard deviations (in percent correct) for each age group in each Planar Orientation by Filtering condition separately. (DOCX) [file pone.0138812.s001.docx]

**S1 Table. Mean and standard deviation of accuracy (in percent correct) for each age group in each Planar Orientation by Filtering condition separately.**

| **Age** | **Upright-HV** | | **Upright-H** | | **Upright-V** | | **Inverted-HV** | | **Inverted-H** | | **Inverted-V** | |
| --- | --- | --- | --- | --- | --- | --- | --- | --- | --- | --- | --- | --- |
|  | ***Mean*** | ***std*** | ***Mean*** | ***std*** | ***Mean*** | ***std*** | ***Mean*** | ***std*** | ***Mean*** | ***std*** | ***Mean*** | ***std*** |
| **6-7** | 83.8 | *5.7* | 81.8 | *6.1* | 68.8 | *9.3* | 73.7 | *8.1* | 71.9 | *7.6* | 71.3 | *7.6* |
| **8-9** | 88.1 | *6.4* | 88.3 | *7.1* | 78.4 | *9.1* | 86.4 | *4.1* | 85.4 | *8* | 79.4 | *8.7* |
| **10-11** | 93.3 | *3.7* | 91.6 | *4.9* | 86.6 | *5.9* | 88.6 | *5.7* | 88.9 | *6.4* | 87.7 | *6.2* |
| **12-13** | 95.3 | *3.5* | 94.8 | *4.6* | 90.3 | *5.7* | 93.5 | *4.2* | 91 | *6.2* | 90.4 | *6.2* |
| **14-15** | 93.3 | *4.7* | 92.7 | *5.7* | 88 | *7.8* | 91.8 | *5.6* | 90.6 | *6.5* | 88.7 | *7.5* |
| **16-17** | 93.3 | *4.6* | 93.4 | *5.6* | 89.6 | *5.1* | 91.6 | *6.1* | 91.6 | *5.9* | 89.9 | *5.5* |
| **18-19** | 92.6 | *4.9* | 92 | *5.1* | 88.8 | *6.8* | 93 | *3.3* | 92.7 | *4.2* | 89 | *5.9* |
| **20-35** | 95.7 | *4.4* | 94.9 | *5.6* | 91 | *4.9* | 94.2 | *3.5* | 93 | *4.7* | 92.6 | *5* |
| **59-74** | 92.8 | *2* | 92.1 | *2.4* | 87.1 | *2.1* | 84.3 | *1.5* | 84.7 | *3.5* | 87 | *2.8* |
| **Total** | 92.3 | *5.5* | 91.5 | *6.3* | 85.9 | *9* | 89.1 | *7.5* | 88.3 | *8.2* | 86.7 | *8.5* |
