# Supplementary material for: Selectivity of Face Perception to Horizontal Information over Lifespan (from 6 to 74 Year Old)
Source: PLoS One. 2015 Sep 23;10(9):e0138812. doi: 10.1371/journal.pone.0138812 (PMC4580649; doi:10.1371/journal.pone.0138812)
Supplement: S1 Text — (DOCX) [file pone.0138812.s002.docx]

**S2 Text. Age influence in filter by planar orientation (upright versus inverted) conditions.**

*GLM analysis of upright and inverted RT*

Individual correct RT were submitted to a GLM analysis with Planar Orientation (upright versus inverted) and Filter type (HV, H and V) as categorical within-subject predictors and age (log of age in months) as a continuous between-subject predictor. GLM revealed main effects of planar orientation (F(1,280)=14.2, p< .0002, η^2^= .0018) and a significant interaction between planar orientation and age (F(1,280)=22.5, p< .00001, η^2^= .0016). The triple interaction between planar orientation, filter type and age was significant (F(1,280)=4, p< .05, η^2^= .0005).

We explored the triple interaction further by running separate GLM analyses per Filter type. When faces were horizontally-filtered (i.e., H condition), the main effect of Planar orientation was significant (F(1,280)= 7, p< .009, η^2^= .0012) and was qualified by a significant interaction with age (F(1,280)= 19.3, p< .0001, η^2^= .003). When faces contained both horizontal and vertical information (i.e., HV condition), the main effect of age and its interaction with Planar Orientation were significant (F(1,280)= 5.9, p< .016, η^2^= .01 and F(1,280)= 10.2, p< .0016, η^2^= .002, respectively). In contrast, there was no significant main effect nor interaction on correct RT when faces were vertically-filtered (Fs< 3, ps> .08, η^2^< .004).

*Partial correlation analyses of upright face-specific processing*

We ran two-tailed Pearson partial correlation analyses to capture the linear function relating age to face-specific processing in HV, H and V orientation bands. We evaluated the relationship between age (log-transform of age expressed in months) and upright performance whilst removing variance associated with inverted performance in each filter condition separately. This analysis highlights the development of face-specific processes selectively engaged for upright faces when the variance due to general, cognitive mechanisms engaged for inverted faces is removed.

Upright performance in HV and H conditions correlated significantly with age when the respective inverted performance was controlled for (HV: r= -0.24, 95% CI=[-0.36 -0.12], p< .0001; H: r= -0.27, 95% CI=[-0.39 -0.15], p< .0001). Correlation coefficients were negative as upright RT got faster as a function of age. The correlation coefficient in the V condition (r= -0.12, CI=[-0.24 0.004], p= .05) covered 0 and was marginally smaller than the correlation coefficients estimated in H condition (p= .055, Steiger, 1980). This confirms GLM results that the evolution of face-specific processing as a function of age was most robust in the former than the latter case.
