# Supplementary material for: Selectivity of Face Perception to Horizontal Information over Lifespan (from 6 to 74 Year Old)
Source: PLoS One. 2015 Sep 23;10(9):e0138812. doi: 10.1371/journal.pone.0138812 (PMC4580649; doi:10.1371/journal.pone.0138812)
Supplement: S2 Text — (DOCX) [file pone.0138812.s003.docx]

**S3 Text. Analyses of the horizontal advantage as a function of age.**

Previous studies showed that identity processing in adults is better when it is based on horizontal than vertical face information for upright but not for inverted faces. This face-specialized horizontal tuning is masked by our FIE analyses. For sake of comparability with previous works, we measured the horizontal processing advantage in upright and inverted condition at the individual level based on a horizontal advantage ratio index (i.e., (vertical RT – horizontal)/ (vertical RT + horizontal); Figure S3). One-tailed t-tests were used to test the hypothesis of a positive horizontal advantage.


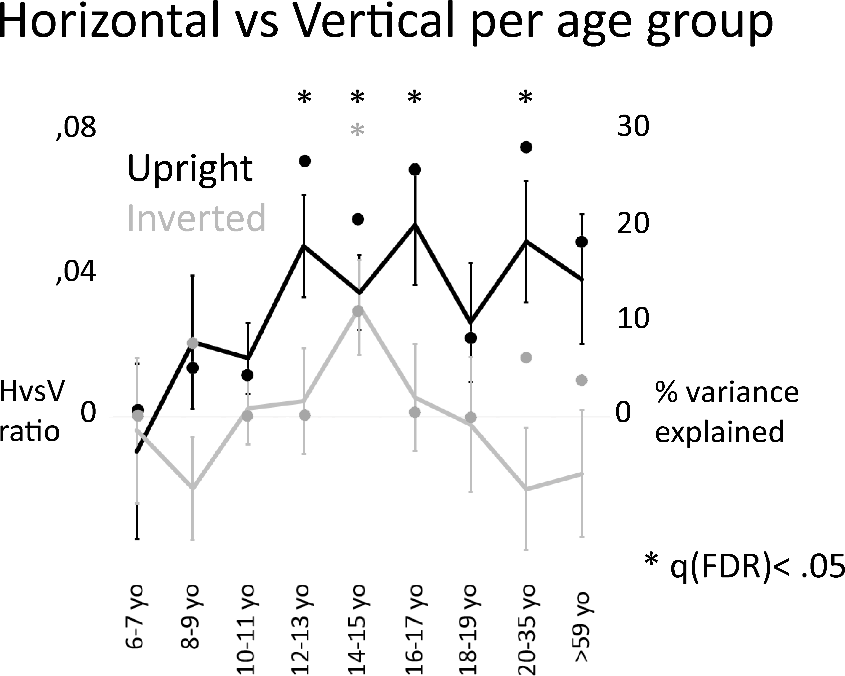


Figure S3. Horizontal advantage ratio mean and size. Mean horizontal advantage ratios (in RT) in the different age groups are plotted separately for upright and inverted conditions. Lines illustrate the mean horizontal advantage ratio per age group. Error bars represent standard errors of these means. Circles depict the size of the horizontal advantage (in terms of percentage of variance explained) for upright and inverted conditions.

Figure S3 depicts the average horizontal advantage ratio indices of each age group in upright and inverted conditions. For the processing of upright faces, the horizontal advantage was significant between 12 and 18 year-old (qs(FDR)< .009) and in young adulthood (qs(FDR)< .02). For inverted faces, no horizontal advantage was expected and this was confirmed in all age groups (qs(FDR)> .2) except the 14-15 year-old group which showed a significant horizontal advantage (q(FDR)< .05). Still the horizontal advantage at 14-15 year-old was twice larger in upright than inverted condition (Figure S3).

In previous works, it was proposed that the specialization of face processing may reside in its peculiar reliance on the horizontal information conveyed by upright faces. We therefore hypothesized that horizontal performance advantage in upright but not inverted condition should be a reliable predictor of face-specialized processing. We tested this prediction directly by performing a two-tailed partial correlation between FIE in HV condition, taken as a proxy of the specialized processing of broadband face information, and upright horizontal advantage while controlling for inverted horizontal advantage. This partial correlation was robust (r= .24, CI= [.1108.3713], p<. 00001) confirming Pachai et al. (2013) finding that horizontal tuning is a reliable predictor of FIE size.
